# Assessment of Groundwater Quality in the Talensi District, Northern Ghana

**DOI:** 10.1155/2020/8450860

**Published:** 2020-04-10

**Authors:** Larry Pax Chegbeleh, Bismark Awinbire Akurugu, Sandow Mark Yidana

**Affiliations:** ^1^Department of Earth Science, University of Ghana, Box LG. 58, Legon, Accra, Ghana; ^2^Council for Scientific and Industrial Research-Water Research Institute, Box M 32, Accra, Ghana

## Abstract

A comprehensive chemical quality assessment of groundwater resources in the Talensi District has been conducted using conventional graphical methods and multivariate statistical techniques. The study sought to determine the main controls of groundwater chemistry and its suitability for domestic and irrigation purposes in the district. Silicate and carbonate mineral weathering were identified as the main controls on groundwater chemistry in the district, with reverse ion exchange also playing a role. High nitrate and lead levels observed have been associated with agrochemicals and wastewater from farms and homes. Three main flow regimes have been identified with Q-mode cluster analysis, in which mixed cation water types have been revealed, where areas designated as recharge zones are dominated by Na^+^ + K^+^–Mg^2+^–HCO_3_^−^ fresh water types characterised by low mineralisation and pH, which evolve into Mg^2+^– Na^+^ + K^+^– HCO_3_^−^ fresh water type with corresponding increased mineralisation of the groundwater. Based on the water quality index (WQI) technique modified for the district and an interpolation technique using ordinary kriging developed from a well-fitted exponential semivariogram for the estimated WQIs, the groundwater quality has been spatially classified as generally ‘good' to ‘excellent' for domestic purposes. Generally, the quality of groundwater for domestic usage deteriorates as one moves towards the north of the district, whereas waters in the east and west present the best quality. Classifications based on the United States Salinity Laboratory (USSL), Wilcox, and Doneen diagrams suggest that groundwater from the unconfined aquifers of the district is of excellent quality for irrigation purposes.

## 1. Introduction

An overview of rural and small-town water services reported by the Community Water and Sanitation Agency (CWSA) [1] suggests that groundwater remains the most important source of fresh water supply for mainly domestic, agricultural, and industrial purposes in the semiarid climates of northern Ghana. Talensi District is one of such places where the populace relies on groundwater through boreholes and open wells for mainly their potable water and other domestic and agricultural related water needs. The presumed resilience of groundwater to climate change and variability, evaporative losses, and protection from common sources of pollution make the resource a preferred source of drinking water supply, since in most cases little or no treatment is required prior to consumption as compared to surface water sources [2]. For the people of Talensi District and several other districts in northern Ghana, the reliance on groundwater for various purposes is not a question of safety but availability and convenience, as the alternative is resorting to other sources of water such as rivers, dams, streams, ponds, and many more, some of which are deemed unwholesome for such purposes [3].

Several combinations of factors work together and/or in isolation to determine the quality of groundwater in space and time. Therefore, groundwater is not always as safe as it is believed to be, since its quality can be influenced negatively by weathering of the rock matrix through which it travels [4–6]. The degree of rock weathering which influences groundwater quality is also dependent on factors such as the residence time, pH, and ambient temperatures [7]. For instance, the dissolution of “hard” rocks and associated silicate minerals is a very slow process, aided by high temperatures and low pH. The influence of rock weathering on groundwater quality has been extensively documented in Ghana [6–12]. Furthermore, population growth, unplanned developments, and other anthropogenic activities have also been identified in the literature as sources of groundwater degradation [11, 13–15]. Trace elements, nutrients, and pesticides in rivers have been reported to seep into and contaminate groundwater [16]. Contamination of groundwater through anthropogenic activities is more likely to occur in shallow unconfined aquifers as compared to deep confined aquifers, since the shallow unconfined aquifers are superficially situated relative to the ground surface and thus susceptible to attacks by surface to near-surface contaminants of varied sources. Therefore, in places where groundwater recharge zones and surface activities are not protected and regulated, respectively, groundwater could be severely contaminated and, given its architecture, cost a fortune to remediate [7, 17].

The problem with groundwater contamination is that there are other indirect pathways by which the contaminants could end up in humans besides the direct consumption of contaminated groundwater. For instance, contaminated groundwater used for irrigation of crops or for animal consumption could end up as a public health hazard when these farm products are consumed. Therefore, it is imperative to holistically assess the parameters that affect the quality of groundwater as well as identify the sources of these contaminants as a means of sustainable management of the resource [18, 19].

A combination of geostatistical and conventional hydrochemical plots has been at the forefront to this end. Eneke et al. [14] adopted geostatistical and conventional hydrochemical techniques in an attempt to identify the main factors controlling groundwater chemistry in Douala, Cameroon, an area characterised by rapid urbanisation and industrialisation. The study revealed that groundwater in the area is acidic (pH between 4.1 and 6.9) and the groundwater quality was mainly controlled by anthropogenic activities. Adopting a similar methodology, Yidana et al. [7] used a factor model to examine the main hydrochemical processes that influence fluoride and other major ion variations in Savelugu, northern Ghana. Silicate mineral weathering, dissolutions of soluble salts, oxidation reactions, and dissolutions of sulphate minerals were found to be the four main factors controlling the hydrochemistry of groundwater resources in the area. In an effort to characterise the suitability of groundwater resources of northern Ghana for domestic and agricultural uses, Anku et al. [13] analysed groundwater samples from the underlying fractured aquifers. Results showed that pH values range from slightly acidic to slightly basic, with electrical conductivity (EC), total dissolved solids (TDSs), calcium, magnesium, and sodium values being below WHO [20] recommended standards for potable water. Spatial distribution maps also revealed pollution with nitrate in the western portions of the study area, which were attributable to anthropogenic activities. Hence, the present study adopts similar techniques to assess groundwater quality for domestic and irrigation purposes and the main factors controlling the hydrochemistry of groundwater in the Talensi District in northern Ghana.

## 2. Study Area

### 2.1. Location, Topography, and Climate

**Figure 1:**
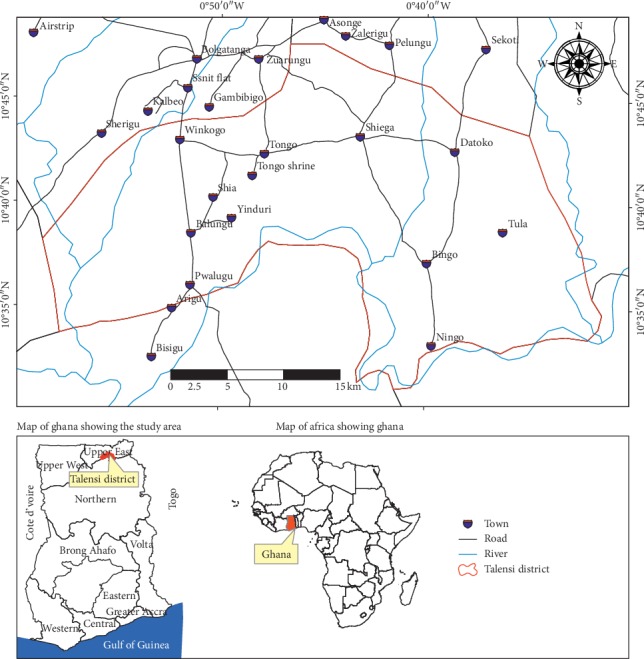
Map of Ghana showing the study area.

The study was conducted in the Talensi District, in the Upper East Region, Ghana. The district lies within the boundaries of longitudes 0°31′ and 1°05′ west and latitudes 10°35′ and 10°60′ north. It shares boundaries with Bolgatanga Municipal to the north, west, and east Mamprusi Districts to the south, Bawku West District to the east, and Kassena-Nankana District to the west (Figure 1). It has a total land area of about 838 km^2^ and population size of 81194 people with a population density of 98.8 persons per kilometre square [21]. The majority of the inhabitants (about 90%) in the district are peasant farmers. Small-scale mining, artisanal stone crushing, agroprocessing, charcoal burning, firewood harvesting, and irrigation farming constitute the other sources of income in the district [3].

The topography of the district is generally flat with relatively undulating lowlands, with gentle slopes ranging between 1% and 5%. Elevations range between 100 m and 200 m, with some isolated rock outcrops and uplands mainly around the district capital Tongo and Yinduri reaching 400 m high. The district is drained by several rivers and tributaries during the rainy season, but in the dry season, most of the tributaries dry out leaving only portions of the Red and White Volta Rivers flowing through the eastern and southern boundaries of the district. The White Volta River meanders in and out of the district until it flows down towards the southwestern part of the district.

The district lies in the Savannah zone, characterised by semiarid conditions with a single rainy season which runs from May to October. The rest of the months of the year is characteristically dry, with barely any rains. Recent annual rainfall records (2008–2017) from the Ministry of Food and Agriculture (MOFA) suggest yearly rainfall ranges between 503 and 997 mm, with an annual average of 837 mm [22]. The area is almost always relatively warm, with temperatures reaching 45°C in March and April and recording a minimum of 12°C in December [3]. The prolonged dry season is accompanied by very low relative humidity which reaches 10% during harmattan (the dry and hazy northeast trade wind) in December and January, and with the onset of the rains, it rises gradually up to 65% maximum [23].

### 2.2. Geology and Hydrogeology

**Figure 2:**
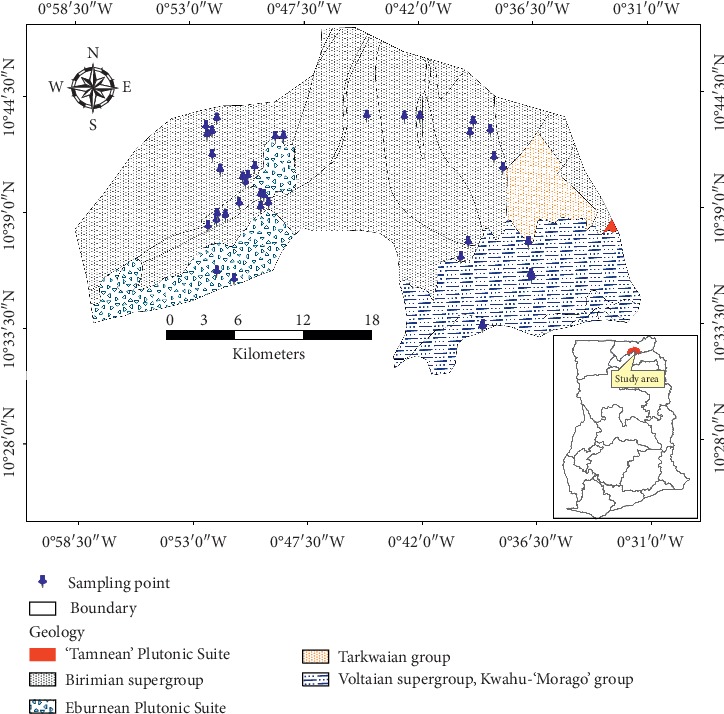
Geological map of the study area.

A large portion of the district (about 75%) is underlain by crystalline Precambrian rocks of the Birimian supergroup, which is intruded by granitoids of the Eburnean and Tamnean Plutonic Suites (Figure 2). The Birimian typically consists of gneiss, phyllite, schist, migmatite, granite-gneiss, and quartzite, whereas the associated intrusions are composed mainly of K-feldspar-rich-granitoids such as granite and monzonite (Bongo type) [24]. The lower and eastern portions of the district are underlain by the Voltaian supergroup (Kwahu-Bombouka group), which is composed mainly of fine-grained micaceous sandstones, medium-grained mudstones, and shales, and the Tarkwaian. They overlie the Birimian and Tarkwaian in the district and are thought to be of late Precambrian to Paleozoic age. The Voltaian supergroup is subdivided into three main stratigraphic units based on lithology and field relationships: Basal sandstones, Oti/Pendjari, and Obosum supergroups. The Basal sandstones comprise quartz sandstone formation of about 75 m thick and commonly occur along the northern and western edges of the Voltaian supergroup. The Oti/Pendjari, on the other hand, is much thicker (1500 m–4000 m) and comprises argillaceous sandstones, arkose, siltstones, interbedded mudstone, sandy shale, and conglomerates [25]. The Obosum formation of the Voltaian supergroup consists of dirty-yellow, fine-grained, thinly bedded, and micaceous feldspathic quartz sandstones with subordinate argillite intercalations and whitish-yellow, massive, fine- to medium-grained, cross-bedded arkosic, and quartzose sandstones.

**Figure 3:**
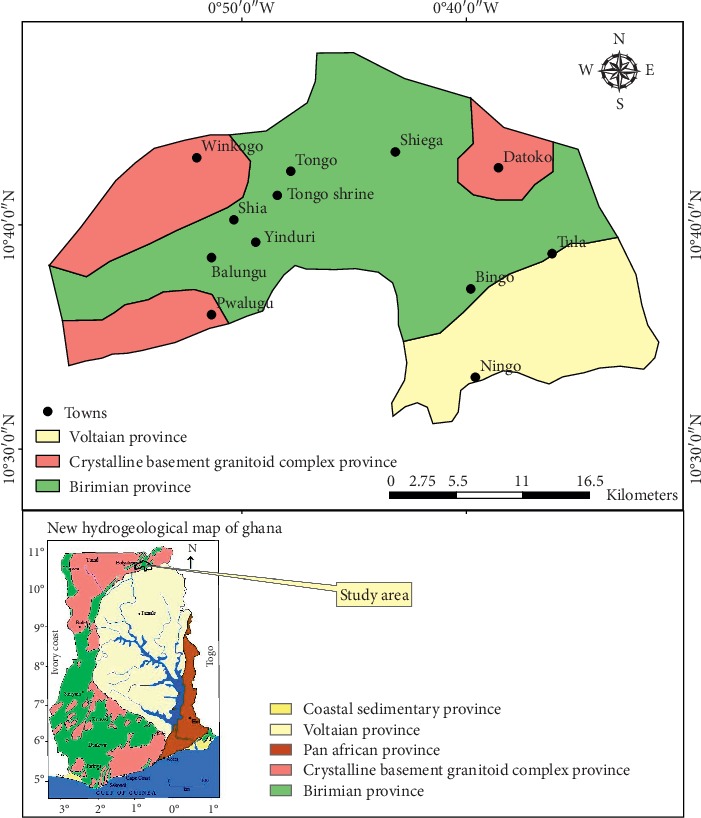
Hydrogeological map of the study area modified from the new hydrogeological map of Ghana [27].

The district falls within three main hydrogeological provinces: the Voltaian, Crystalline Basement Granitoid Complex and Birimian Provinces [26] (Figure 3). Generally, the Birimian rocks exhibit deeper weathering than the granitoids. Groundwater occurs mainly within the saprolite, saprock, and fractured bedrock within the Birimian Province. However, the lower and upper parts of the saprolite and saprock, respectively, are the most productive zones in terms of groundwater delivery and normally complement each other in terms of permeability and groundwater storage [28].

Three main types of basement aquifers have been identified: the weathered rock (fracture-related), quartze-vein, and the unweathered fractured aquifers [29]. Banoeng-Yakubo et al. [27] further emphasize that the saprolitic zone results from a combination of the topsoil, underlying lateritic soil, and the variably weathered zone in the Birimian Province. Borehole depths arrange Birimian and the Tarkwaian range between 35 and 55 m with an average of 42 m [30]. Carrier et al. [28] also recorded borehole depth in a similar range of 35 and 55 m with an average of 50 m in the granitoids. Akurugu et al. [2] indicate that the depth to the water table, at the time sampling data were collected for this study, ranges between 2 and 13 m with an average of 8 m. The highest elevations are in the eastern and southeastern sections of the district; as such, groundwater flow follows a general northeast-southwest pattern with a few local flow systems, which cannot be clearly defined [2]. The aquifer hydraulic conductivity has been reported to be highly variable, ranging between 0.001 m/d and 58.396 m/d [2]. The productive zones of the Birimian Province have also been reported to range between 0.2 m^2^/d and 119 m^2^/d, with an average of 7.4 m^2^/d [27].

The Kwahu-Morago of the Voltaian supergroup is a good aquifer zone composed mainly of the sandstone formation. The Voltaian supergroup is characterised by little or no primary porosity; as a result, groundwater occurrence in the area is controlled by secondary porosities from the weathering and fracturing of the rock which has enhanced the storage and transmissive properties of the rocks to form groundwater reservoirs [31, 32].

### 2.3. Land Use/Land Cover Distribution and Soil Types

**Figure 4:**
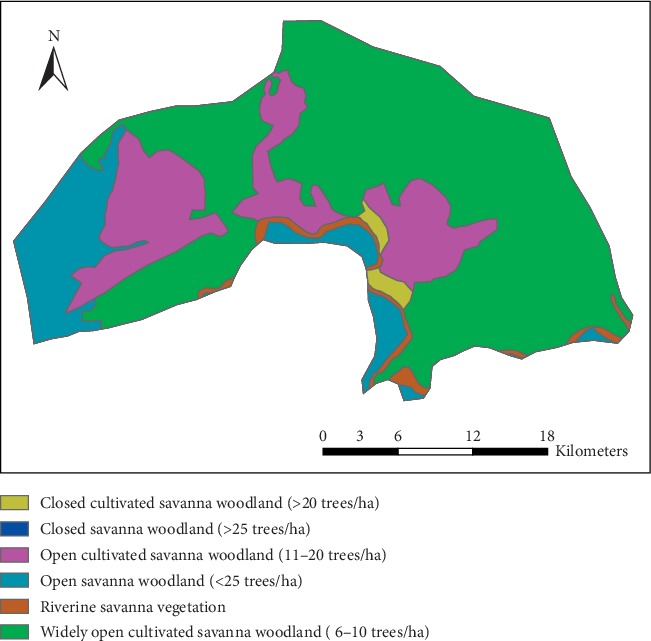
Vegetation cover map of Talensi District.

The vegetation of the district is a typical guinea Savannah woodland, comprising mainly of short widely spread plants and shrubs (Figure 4), which usually shed foliage at the end of the growing season, and trees and ground flora of grass, which get burnt by fire or the scorching sun during the long dry season [3, 33]. Typical trees are dawadawa, shea trees, acacias, and baobab. These conditions impact negatively on rainfall amount in the area, thereby affecting the amount of groundwater. As a predominantly agricultural economy, the excessive temperatures and long dry season periods compel indigents to seek alternative livelihoods elsewhere, usually southern Ghana, in order to cater for the food gap during those periods, which have a tendency of drawing the young and energetic farm labour from the communities [3, 33].

**Figure 5:**
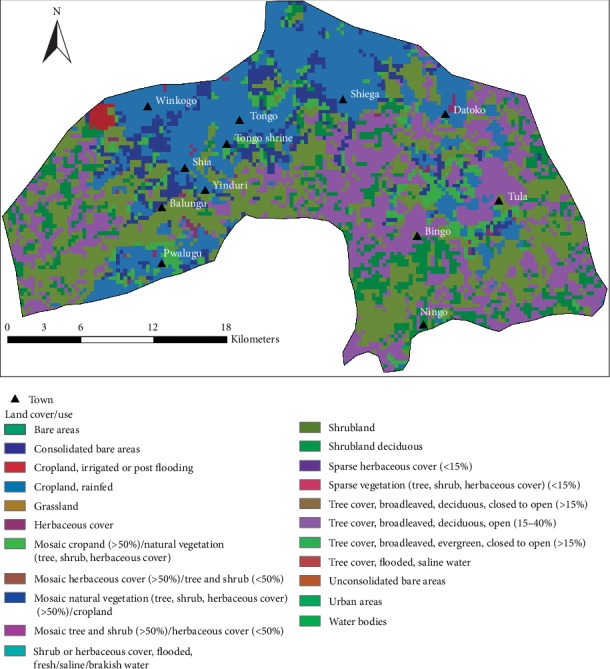
Land cover/use map of Talensi District.

Investigation of nature at which land is put to use by various anthropogenic activities is a key to understanding the quality of groundwater in a given area [34, 35]. In this present study, Land Cover satellite data for the year 2015 was downloaded from the Climate Change Initiative- (CCI-) Land Cover (LC) project which delivers global LC maps at a spatial resolution of 300 m [36]. The data was further processed in ArcGis 10.2 from which various Land Use/Land Cover (LULC) classes of interest that have a significant impact on the general quality of groundwater in the area were identified and delineated (Figure 5). Figure 5 provides a confirmatory picture of LULC of the study area as corroborated by the assertion that the entire area is largely rural, comprising rain-fed croplands, mosaic trees and shrubs, patches of bare land, etc. The LULC system in the district and Ghana as a whole is usually intercalated, with no well-defined pattern. A few urban-like areas include Tongo, Pwalugu, and Winkogo located in the central, northwestern, and southwestern parts of the district (Figure 5).

**Figure 6:**
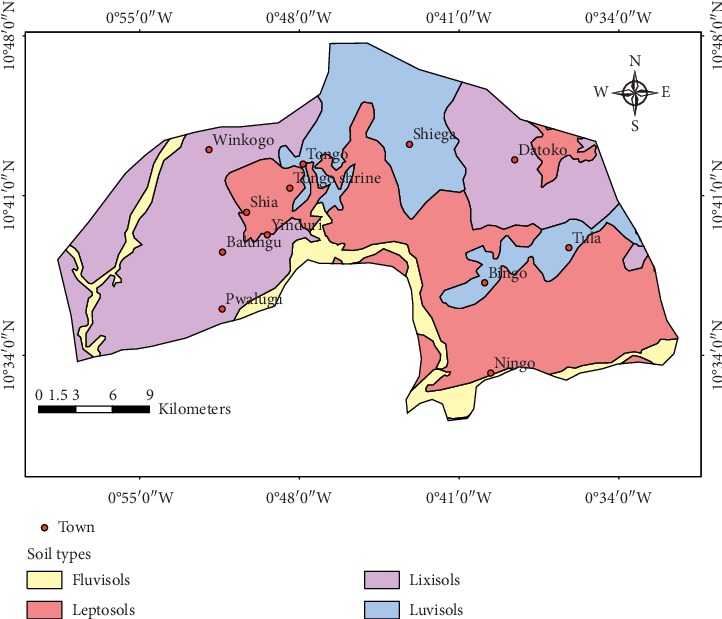
Map of soil types in the district.

Soil serves as the natural medium through which plants grow on land. As such, it is arguably one of the most important natural resources of a nation. Different types of soils with varying suitability for different purposes exist. The bedrock, relief, drainage, climate, microorganism, and several other factors determine the type of soil that exists in a particular locality. The district is dominated by lixisols, leptosols, and luvisols (Figure 6), mainly due to these reasons. Obeng [37] suggests that soils of the Savannah belts are characteristically low in organic matter and soil moisture within the surface horizon mainly as a result of the dominant grass vegetation and low and variable rainfall patterns. As a result, the soils are generally prone to erosion and declining fertility, when subjected to even the least negative land use practice.

## 3. Methodology

Groundwater samples within the study area were collected through boreholes and hand-dug wells (ranging between depths of 35 and 84 m) in the month of November 2017, in the dry season, when water resources in the region are expected to run low [2]. Samples were collected so as to represent the domain as evenly as possible by taking at least one or two samples from communities with boreholes and/or wells. Prescribed standard protocols for water sampling and storage [38–40] for various purposes were adopted throughout the study. A total of thirty-nine groundwater samples were collected (26 boreholes and 13 hand-dug wells) and analysed for major ions and trace elements. Boreholes were first purged for about five to ten minutes to rid them of stagnant waters (usually until when EC/pH was stable) before sampling was done. Physical parameters such as pH, Electrical Conductivity (EC), Total Dissolved Solids (TDSs), and temperature were measured in situ using HI98129 Low Range pH/Conductivity/TDS Tester. The water samples were filtered through a 0.45 *μ*m cellulose acetate membrane and collected in 250 ml sterilized low-density polyethylene bottles in two sets: a bottle containing nonacidified samples for major anions analysis and the other bottle containing samples for major cations and trace element analysis acidified with concentrated nitric acid (HNO_3_) to a pH less than two, to prevent precipitation of the metals, oxidation reactions, and absorption to container walls and to reduce microbial activity [41]. All the samples were clearly labelled and preserved in ice chests that had been conditioned to a temperature of about 4 °C by ice until they were ready to be transported to the laboratory for analysis. Water samples were analysed for physicochemical parameters such as pH, electrical conductivity (EC), calcium (Ca^2^⁺), magnesium (Mg^2^⁺), sodium (Na⁺), potassium (K⁺), sulphate (SO_4_^2−^), chloride (Cl⁻), bicarbonate (HCO_3_^−^), nitrate (NO_3_^−^), phosphate (PO_4_^3−^), fluoride (F^−^), salinity (SAL), and Total Dissolved Solids (TDSs) in the laboratories of the Chemistry Department of National Nuclear Research Institute (NNRI) of the Ghana Atomic Energy Commission (GAEC). Physical parameters such as pH, EC, TDS, and salinity were measured using HI 2550 pH/ORP & EC/TDS/NaCl Meter. Alkalinity, Ca^2^⁺, and total hardness (TH) were determined by titrimetric methods. Atomic absorption spectroscopy (AAS) was used to determine the major cations and trace elements. Anions such as Cl^−^, SO_4_^2−^, NO_3_^−^, F^−^, and PO_4_^3−^ were analysed using Dionex DX 120 ion chromatograph.

The dataset was subjected to charge balance error (CBE) to test the accuracy of the analysis (equation (1)). A charge balance error value of ±5% and below is generally acceptable and shows that the analysis of the parameters shows a good balance of the cations and anions [42].

(1) $$\begin{array}{c} \text{CBE}=\frac{\sum m_{c}\left|z_{c}\right|-\sum m_{a}\left|z_{a}\right|}{\sum m_{c}\left|z_{c}\right|+\sum m_{a}\left|z_{a}\right|}\times 100, \end{array}$$

where *m*_*c*_ and *m*_*a*_ and *z*_*c*_ and *z*_*a*_are, respectively, molar concentrations of major cations and anions, and charges of cations and anions.

The datasets were equally subjected to normality test, since some of the statistical techniques such as cluster analysis assume a normal distribution. Datasets that were not normally distributed were log-transformed and/or standardized to their *z*-score values (equation (2)):

(2) $$\begin{array}{c} z=\frac{x-\mu }{s}, \end{array}$$

where *z*, *x*, *µ*, and *s* are z-score, sample value, mean, and standard deviation, respectively.

Q-mode and R-mode hierarchical cluster analyses (HCAs) were performed using the transformed dataset. Q-mode and R-mode HCAs split samples and parameters, respectively, into groups/clusters based on some similarities/dissimilarities. The Q-mode HCA is used to assess spatial associations or evolution of groundwater in space and/or time, whereas R-mode HCA is used to determine and rank the sources of variation in the hydrochemistry. The squared Euclidean distance and Ward's agglomeration method were employed in this study, since a combination of these two has been identified to yield optimal results in HCAs [43, 44], although several similarity/dissimilarity and agglomerative techniques are available in HCAs. Ternary diagrams and water quality assessment and classification plots such as Piper [45], Wilcox [46], water quality index (WQI), and United States Salinity Laboratory [47] were used in conjunction with the geostatistical techniques to characterise and classify groundwater quality in the district for domestic and irrigation purposes.

Nevertheless, water that is deemed fit for one purpose may be undesirable for another, as such an integrated approach which incorporates all the relevant chemical parameters of interest, usually guided by WHO [20], and local drinking water standards in a particular groundwater system is relevant in characterising the suitability of water domestic for purpose. The water quality index (WQI) has been adopted by many studies for such purposes [48–52]. The same has been adopted in this study, the weighted arithmetic index approach modified after Brown et al. [53], to assess the quality of groundwater for drinking purposes. This method involves assigning weights (wi) to water quality parameters based on their health implications in potable water. Parameters such as Pb, F-, NO_3_^−^, and pH which are believed to be of critical health importance and significant impactors of the quality of groundwater were assigned the maximum value of 5. TDS, TH, Ca^2+^, Mg^2+^, Na^+^, K^+^, CI^−^, SO_4_^2−^, PO_4_^3−^, and Fe were assigned values of 2 to 4 (Table 1) depending on their perceived significance relative to groundwater potability and health implications. This method also involves the computation of relative weights (*Wi*) (equation (8)) and quality rating scale (*qi*) (equations (4)) and determination of the subindex (SI) (equation (5)) and water quality index (WQI) (equation (6)). The computed WQIs were then classified according to Sahu and Sikdar [54] (Table 2).

(3) $$\begin{array}{c} Wi=\frac{wi}{\sum wi}, \end{array}$$

(4) $$\begin{array}{c} qi=\frac{Ci}{Si}\times 100, \end{array}$$

where *Ci* and *Si* are parameter concentration and objective to be met, respectively.

(5) $$\begin{array}{c} \text{SI}=Wi\times qi, \end{array}$$

(6) $$\begin{array}{c} \text{WQI}=\sum_{n=1}^{n}\text{SI}. \end{array}$$

The assessments of groundwater quality for irrigation purposes in this study are mainly sodium-based/related techniques, which compare the concentration of Na^+^ to other ions in the groundwater system. Relatively high levels of Na^+^ as compared to other cations such as Mg^2+^ and Ca^2+^ tend to reduce soil permeability which results in poor soil structure for drainage [55, 56] since, through ion exchange, Na^+^ tends to get absorbed to surfaces of clay materials and displace Ca^2+^ and Mg^2+^ in solution. These conditions lead to a soil type that is unsuitable for optimal crop growth and production. The sodium adsorption ratio (SAR) is one of such methods used for irrigation water quality assessment. It is an index that measures the relative content of sodium to the sum of calcium and magnesium in water used for irrigation (equation (7)) whereas EC is used as a yardstick to measure the salinity of the water.

(7) $$\begin{array}{c} \text{SAR}=\frac{\text{Na}}{\sqrt{\left(\text{Ca}+\text{Ma}/2\right)}}, \end{array}$$

where concentrations of all ions are in meq/l.

Similarly, the Wilcox [46] diagram was also used to assess the quality of groundwater for irrigation purposes, by plotting the percent of sodium in the water versus salinity. The percent of sodium is calculated as a fraction of the concentration of the total major cations in the water (equation (8)). The Wilcox diagram just like the USSL diagram categorises irrigation water into various suitability ranges based on sodium and salinity hazards.

(8) $$\begin{array}{c} \text{Na}\%=\frac{\text{Na}}{\text{Na}+\text{Ca}+\text{Mg}+\text{K}}\times 100\text{,} \end{array}$$

where concentrations of all ions are in meq/l.

## 4. Results and Discussions

### 4.1. Statistical Summary of Hydrochemical Data

**Figure 7:**
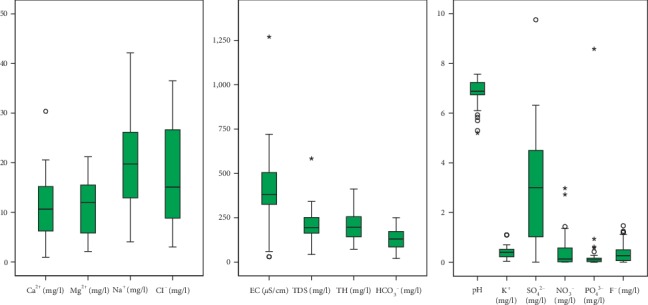
Box-and-Whisker plots for physicochemical parameters used for the study.

The statistical summaries of physicochemical parameters of 39 groundwater samples from wells used in the study are presented in Table 3 and Figure 7. The distribution of most of the parameters in the district is highly variable, suggesting that diverse processes control these parameters in the district.

The values of temperature in groundwater of the study area range from 28.7°C to 34.4°C with a mean value of 31.6 °C, which fall within the ranges of natural water bodies (WHO) [20].

Groundwater pH has been known to influence the dissolution of minerals in a groundwater system as well as affect the quality of water for various purposes. pH in the district does not display much variance; it ranges between 5.2 and 7.6 with an average of 6.8 pH units and a standard deviation of 0.6 (Table 3). Most of the lowest pH values appear to be outliers and extreme outliers (Figure 7); as such, the pH of the groundwater system is almost neutral to closely acidic and falls within the pH ranges for natural waters (4.5–8.5) [57, 58]. About 17% of the water samples fall below the recommended World Health Organisation (WHO) [20] standard (6.5–8.5) for domestic water use. Most of these low pH values occur around Tula and Datuku in the northeastern portions of the district. The recorded low pH values are mainly attributable to CO_2_-charged precipitation and natural biogeochemical processes, plant root respiration and leachates from organic acids from the decay of organic matter [13].

EC and TDS, on the other hand, present the highest variations in the district; EC ranges from 30 *μ*S/cm to 1270 *μ*S/cm with an average value of 403.85 *μ*S/cm (Table 3), which represents fresh groundwater type. The EC values appear to be positively skewed (Figure 7), indicating that the tail is distributed towards the higher values. The EC values have corresponding TDS values ranging from 43 mg/l to 584 mg/l, with an average of 204 mg/l. Generally, EC and TDS values fall within the WHO [20] recommended standard of 2500 *μ*S/cm and 1000 mg/l, respectively, for drinking water. As water from precipitation infiltrates the soil and travels through rock media down the subsurface, it dissolves minerals and carries the dissolved particles along its path. As such, TDS values are usually lowest at points of infiltration, designated as recharge zones, usually with TDS values similar to those of the precipitation in the area, and highest at the points of discharge, thus after it has travelled through the rock media and characteristically dissolved more materials along its path of travels. The dissolution of more minerals by water, therefore, results in the availability of more electrolytes/ions in the groundwater systems and a corresponding high EC value. The high values and extreme outliers in EC values are attributable to the influence of the geology and/or impacts of anthropogenic activities which vary widely in space [7]. The highest EC values occur around Pwalugu and Pusinamoo areas in the southwestern portions of the study area, whilst the lowest values were recorded in the northern and eastern parts of the district.

HCO_3_^−^ also displayed high variance, with a minimum value of 20 mg/l and a maximum of 250 mg/l, giving an average of 125.50 mg/l. HCO_3_^−^ showed a positive linear relationship with pH, as it increased with high pH values and decreased with low pH. In natural water systems, bicarbonate is the dominant anion within pH ranges of 4.5–9 [57]; as such, the dominant anion in the groundwater system was bicarbonate, in the given pH ranges. Although other parameters such as Ca^2+^, Mg^2+^, Na^+^, CI^−^, SO_4_^2−^, NO_3_^−^, and PO_4_^3−^ displayed high variations and deviation from the mean, the values fall within the WHO [59] permissible limits for domestic water use. Most of the parameters appear to be positively skewed and, thus, scaled towards the right tail, except for Mg^2+^, K^+^, and PO_4_^3−^ which appear negatively skewed with outliers skewed to the right (Figure 7).

### 4.2. Main Controls on Groundwater Chemistry

Pearson's correlation coefficients of various analysed parameters were calculated as a basis for making certain inferences and drawing relationships among parameters and, to a large extent, predicting values of other parameters at places without actually measuring them [60]. Pearson's correlation coefficient (*r*) ranges from −1 to 1, where −1 and 1 are perfect inverse correlations and perfect direct correlations, respectively.  Table 4 provides a quick way to identify trends within the water quality parameters. Parameters having correlation coefficient (*r*) of ׀0.5׀ and above are considered to be significantly correlated. The dataset shows that the total hardness (TH) exhibits a significant positive correlation with EC, pH, TDS, Ca^2+^, Mg^2+^, Na^+^, HCO_3_^−^, CI^−^, and salinity (SAL). Calcium and magnesium appear to be the main contributors to TH, stemming from the dissolution of limestone by carbon dioxide-charged precipitation. However, the strong positive correlation of the other parameters with TH suggests a possible contribution of these parameters to the total hardness of the groundwater in the district. Hard water is known to leave scaly deposits in pipes and reduce the cleaning ability of soap and detergents, as well as deteriorate fabrics [61]. Extremely low values of TH are also likely to cause nutrient deficiencies especially of calcium and magnesium; hence, WHO [59] recommends 500 mg/l as the highest permissible limit for TH in drinking water. Similarly, pH also showed a significant positive correlation with TDS, Ca^2+^, Mg^2+^, Na^+^, HCO_3_^−^, and CI^−^. Among these, pH and HCO_3_^−^ showed the highest correlation (*r* = 0.939), with HCO_3_^−^ increasing significantly with increasing pH. The positive correlation with pH suggests the possible release or dissolution of these ions in solutions with changes in pH values. Bicarbonate also relates strongly with EC, TDS, Ca^2+^, Mg^2+^, and Na^+^ (*r* = 0.858, *r* = 0.939, *r* = 0.904, *r* = 0.881, *r* = 0.885, and *r* = 0.668, respectively). The strong correlation with these cations and HCO_3_^−^ suggests a groundwater system possibly dominated by a Ca–Mg–Na–HCO_3_ fresh water type, resulting from the possible dissolution of carbonate minerals such as calcites, dolomites, and aragonite and decomposition of silicate minerals. Although the relationship between F^−^ and other parameters is not well established, it shows a weak positive correlation with EC, TDS, Ca^2+^, Mg^2+^, and HCO_3_^−^ (*r* = 0.327, *r* = 0.322, *r* = 0.369, *r* = 0.289, and *r* = 0.379, respectively) and a strong linear relation with SO_4_^2−^ (*r* = 0.588) (Table 4). This relation implies that F^−^ increases with increasing EC in the district, which agrees with the findings of a research conducted by Yidana et al. [7] in Savelugu and its surroundings, a domain underlaid with geology similar to the current study area.

#### 4.2.1. Hierarchical Cluster Analysis and Hydrochemical Facies

**Figure 8:**
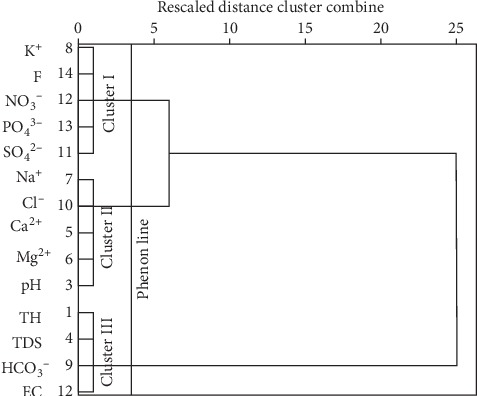
Dendrogram for R-mode cluster analysis.

**Figure 9:**
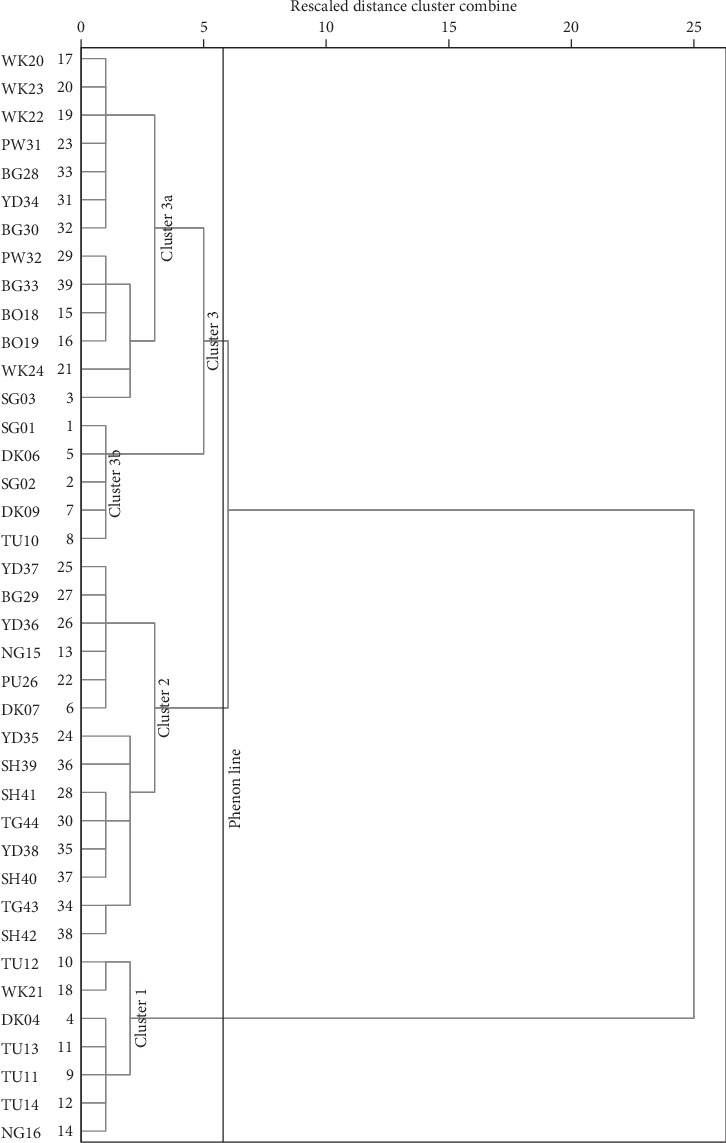
Dendrogram for groundwater spatial associations from Q-mode cluster analysis.

The groundwater samples across the district comprising 39 samples and 14 variables were subjected to hierarchical cluster analysis (HCA). The hydrochemical parameters showed three main cluster groups based on a dendrogram using Ward`s method (Figure 8), with a phenon line drawn at a linkage distance of about 3.5 in R-mode cluster analysis. Cluster analysis places variables/samples into groups based on distinguished similar characteristics and associations with each other, such that the most similar variables/samples are placed in one cluster and connected to a closely related cluster(s) and further from clusters with less relation, all of which are connected to form one big cluster, in agglomerative schedule cluster analysis. Although the definition of clusters based on the dendrogram is subjective, it is, however, informed by the researcher's understanding of the combining environmental factors such as the geology, hydrogeology, and other human activities which prevail in a place and are likely to affect the chemistry of groundwater in the study area [7]. Notwithstanding, the distinction among the groups is clearly shown in the dendrograms (Figures 8 and 9). The variables are clustered into 3 main groups. The dendrogram shows close associations between K^+^, F^−^, NO_3_^−^PO_4_^3−^, and SO_4_^2−^, in Cluster I (CA-I) (Figure 8), which suggests the possible impacts of pollution of infiltrating precipitation and/or recharge, probably from agricultural input fertilizers and related anthropogenic activities. Chemical fertilizers, such as NKP fertilizer, influence groundwater phosphate, potassium, and nitrate content, since these fertilizers are composed mainly of such chemicals, whereas the weathering of K-rich feldspars is associated with the release of potassium and other related ions in solution. Similarly, sulphate could also result from the oxidation of sulphide minerals, especially in recharge areas where the bedrock of the aquifer is exposed to such conditions [62]. The second group which forms Cluster II (CA-II) contains Na^+^, Cl^−^, Ca^2+^, Mg^2+^, and pH and also represents a groundwater system dominated by water-rock interaction [63], probably influenced by acidic groundwater conditions. This interaction is characterised mainly by silicate and carbonate mineral weathering which releases such ions in solution. The third cluster (CA-III) shows the similarity between total hardness (TH), TDS, HCO_3_^−^, and EC. This association suggests the domination of groundwater by precipitation and its associated interaction with atmospheric CO_2_, with total hardness contributing significantly to the EC.

**Figure 10:**
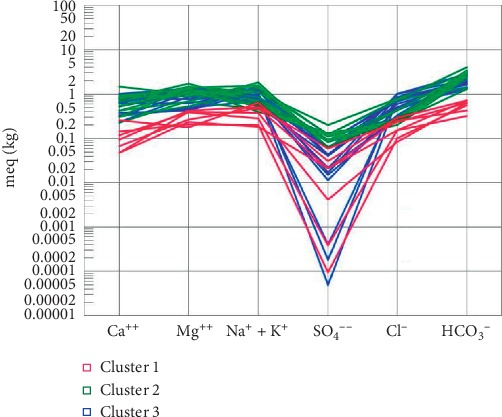
Schoeller diagram made from average concentrations of major ions in the three clusters.

**Figure 11:**
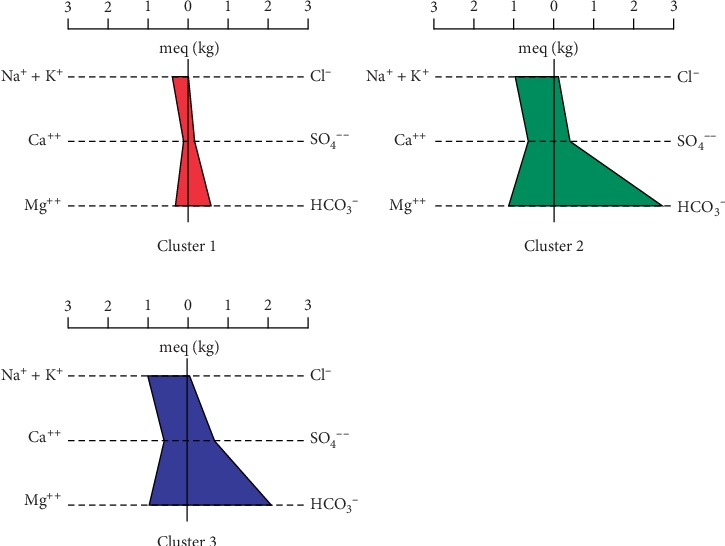
Stiff diagrams made from average concentrations of major ions in the three clusters.

On the other hand, Q-mode hierarchical cluster analysis (HCA) was employed to unveil the spatial relationships in the groundwater parameters, as well as define the flow regimes in various locations in the district and evolutionary sequences along groundwater flow paths as it moves from recharge to discharge zones. Three main spatial groundwater relations have been identified with this method, as illustrated by the three clusters (Cluster 1, Cluster 2, and Cluster 3 (3a and 3b)) (Figure 9). The hydrochemical parameters of the three clusters have been averaged and used to plot Schoeller and Stiff diagrams (Figures 10 and 11) for better visualisation of the main hydrochemical facies and the possible controls of groundwater chemistry represented by these three clusters, for a better understanding of the groundwater flow regime and chemistry in the district. Cluster 1 (CA-1) presents a Na + K–Mg–HCO_3_ fresh water type in the groundwater flow regime with a relatively low average pH of 5.77. The low pH is possibly traceable to the reaction of CO_2_ with precipitation which results in carbonic acid and/or from the reaction of the same when cellular respiration of plants releases CO_2_ into the groundwater system. CA-1 is composed mainly of samples from Tula, Datuku, and Ningo areas which are geographically close together and characterised by the granites, sandstones, limestones, and many more. The first group (Cluster 1) also presents a weakly mineralised groundwater, characterised by relatively lower levels of major ion concentration, which is a characteristic of recharge zones in the flow regime, probably as a result of rapid preferential recharge through macropores or as a result of the short residence time in the geologic material in which it is found. The low pH conditions as illustrated by CA-1 also create a conducive environment for rock mineral weathering, specifically the majority which are silicate minerals and the minority which are carbonate minerals which characterise the geology of the district, which releases ions such as Na^+^, Ca^2+^, and Mg^2+^ in solution.

Conversely, subsequent clusters show relatively moderate to high mineralisation in Cluster 3 (CA-3) and Cluster 2 (CA-2), respectively (Figure 11), suggesting a longer residence time and a higher groundwater–rock interaction as the water travels from recharge areas to discharge zones [56, 57]. The total dissolved ion content seems to increase as the groundwater apparently evolves from a Na + K–Mg–HCO_3_ dominant fresh water type in Cluster 3, identified as intermediary flow zones, to Mg–Na + K–HCO_3_ fresh water type, designated as discharge zones in this study. Inasmuch as an evolutionary sequence has been observed, the groundwater flow regime does not particularly appear to follow the evolutionary sequence as described by Chebotarev [64], which thus suggests a decrease and increase in the concentrations of HCO_3_^−^ and Cl^−^, respectively, in discharge zones, therefore suggesting that there are variable sources of HCO_3_^−^ in the groundwater system in the study area besides precipitation. CA-2 and CA-3 consist of samples mainly located around Winkogo, Balungu, and Pwalugu which are predominately locations with relatively medium to low elevations in the district and are therefore characterised accordingly as discharge zones [57, 64].

The hydrochemistry of the district is unveiled further by constructing Piper [45] and Durov [65] diagrams, although Durov [65] diagram is more advantageous than the Piper diagram, as it further reveals the hydrochemical processes which affect groundwater genesis [66] alongside the water type. Generally, the groundwater is a fresh water type dominated by bicarbonate (HCO_3_^−^ > SO_4_^2−^ + Cl^−^). However, the Piper diagram suggests the presence of chloride and sulphate water types at very low levels. From the Piper diagram (Figure 11), it is apparent that most of the groundwater samples (82%) are dominated by Mg-Ca-HCO_3_ (field I), implying the dominance of alkaline earth over alkali (thus Ca^2+^+Mg^2+^ >Na^+^+K^+^). The remaining 8% of the water samples fall in field IV which represents Na + K-HCO_3_ water type, also signifying the dominance of alkali over alkaline earth; hence, none of the water samples fell in fields II and III in the groundwater system, which signifies Ca-Mg-Cl-SO_4_ and Na-K-Cl-SO_4_, respectively.

**Figure 12:**
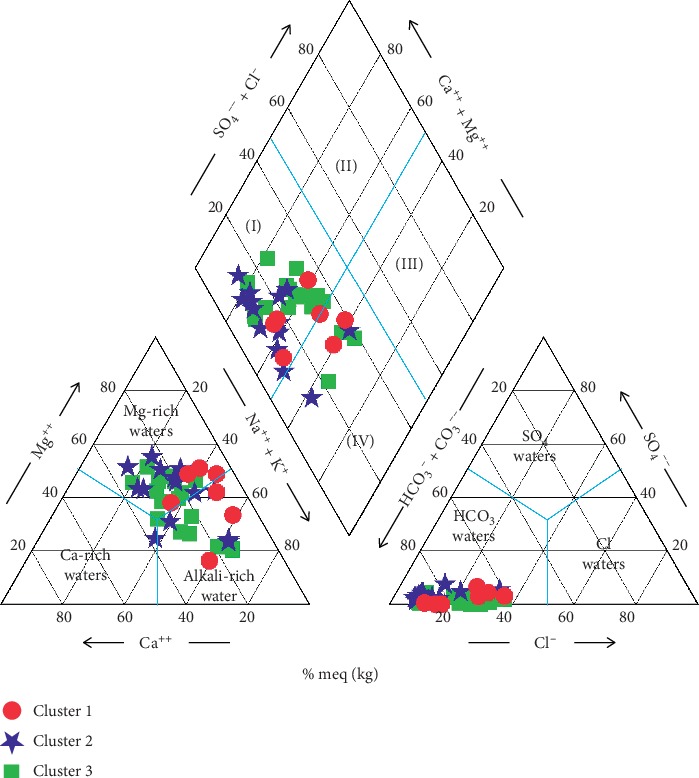
Piper trilinear diagram showing major hydrochemical facies.

**Figure 13:**
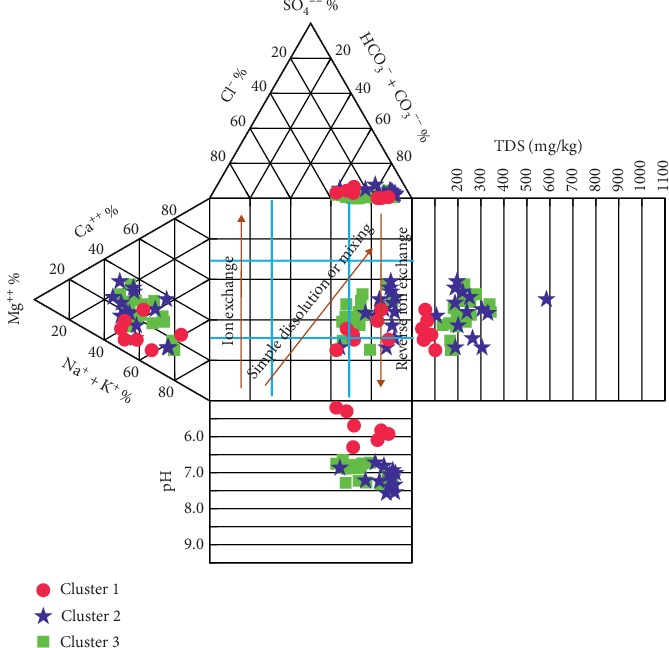
Durov diagram showing hydrochemical facies and processes adapted from Lloyd and Heathcoat [66].

From the Piper plot, it is apparent that Cluster 2 samples are more enriched in Mg and Ca than Clusters 1 and 3 and are the main contributors to the Mg-Ca-HCO_3_ water type identified in the groundwater system of the district. Samples from CA-1 and CA-3, on the other hand, drift more closely to a Na^+^+K^+^ enrichment, with CA-3 being even more so, and can, therefore, be thought of as the main contributor to the Na + K-HCO_3_ hydrochemical facies observed in the Piper trilinear diagram (Figure 12). Durov [65] plot, on the other hand, showed similar hydrochemical facies as the Piper plot, where the cation field is a mixed cation type of water with Cluster 2 being more skewed to Mg-Ca ion dominance whilst Cluster 1 shows enrichment in Na^+^+K^+^ ions (Figure 13). The Durov plot also suggests that simple dissolution and reverse ion exchange be the two main hydrochemical processes affecting groundwater chemistry in the study area. The dissolution of calcite and dolomite is most likely the main contributors of Ca^2+^-Mg^2+^ dominance as exhibited by the cations in the groundwater system. However, the atmospheric deposition of chloride is balanced by a corresponding increase in Na, from the dissolution of albites and orthoclase, which has resulted in the Na^+^+K^+^ prevalence, as exhibited mainly by Cluster 1 samples. The reverse ion exchange is explained by the prevalence of Mg-Ca ions in solution which suggests the replacement of Na^+^ with either Ca^2+^ or Mg^2+^, leading to a groundwater system dominated by these alkaline-earth water types. In a groundwater system dominated by sodium ions, reverse ion exchange could occur in the presence of Ca^2+^ and Mg^2+^, where two sodium ions, usually associated with clay, can replace a calcium or magnesium ion, and in the process, altering the composition of the water. This appears to be a significant process in the groundwater system in the district (Figure 13). Generally, Cluster 1 samples are acidic followed by Cluster 3 and Cluster 2, respectively. Low pH values influence the dissolution of minerals and, therefore, lead to an increase in TDS with a corresponding pH rise. Hence, Cluster 1 samples exhibit the lowest TDS values, a characteristic of fresh water of meteoric origin. Clusters 3 and Cluster 2 are within the intermediate and discharge zones and, therefore, exhibit relatively medium and high pH and TDS, respectively.

#### 4.2.2. Predominant Sources of Variation in Groundwater Chemistry in the Study Area

Principal component analysis (PCA) is a data reduction technique that scrutinises a set of data to unveil the significant principal components (PCs) in the data to aid interpretation of a large series of data and to visualise the correlations between the variables and factors and hopefully be able to limit the number of factors causing variations in the dataset. PCA was performed using a correlation matrix, which brings the measurements onto a common scale, and the main components extracted based on eigenvalues greater than or equal to 1 [67], with the principal components (PCs) being sorted in a diminishing order of variance, such that the most important principal components are listed first. By nature, the factors controlling the hydrochemistry of the groundwater in the district would have some degree of correlation. To ensure that the factors do not correlate with each other and that parameters do not correlate significantly with more than one factor, varimax rotation was used. Varimax rotation produces orthogonal factor rotation, such that the resultant factors are uncorrelated and easily interpretable [7]. Based on the abovementioned categorisation, the final factor model produced three factors that account for more than 72% of the total variance in groundwater hydrochemistry in the district (Tables 5 and 6). Usually, parameters with high communalities are the parameters that contribute significantly to the factors, hence the critical lower limit of 0.5 was set, and the parameters with fewer communalities were excluded. PO_4_^3−^ showed a nonsignificant communality; it was, however, included in the final factor loadings due to its significant loading with one of the factors. Similarly, KMO and Bartlett`s test of sphericity showed that the dataset contained some statistically significant correlation in the correlation matrix, which is in line with the outcome of the Pearson correlation results. Also, the results of the Kaiser-Meyer-Olkin measure of sampling adequacy, which is used to assess whether a dataset qualify to be subjected to PCA and has a critical lower limit of 0.4, gave a value of 0.622 indicating that the data is adequate enough for PCA.  Table 5 summarises the results of the three main principal component loadings for the hydrochemistry of the district. The extracted factor loadings (Table 5) show that Component 1 (PC1) accounts for the highest variance of above 44% and has high factor loadings with EC, HCO_3_^−^, pH, Ca^2+^, Na^+^, Mg^2+^, and Cl^−^. The high component loading of HCO_3_^−^, Ca^2+^, Na^+^, Mg^2+^, and Cl^−^ with PC1 suggests a combined set of factors influencing the hydrochemistry such as intense chemical weathering processes which include the dissolution of silicates and carbonate minerals and contributions from precipitation, in which the assertion corroborates the results of the cluster analysis. Component 2 (PC2), on the other hand, represents about 16% of the total variation in the hydrochemistry and loads significantly with SO_4_^2−^, F^−^, and PO_4_^3−^ which suggests the influence of domestic wastewater and agrochemicals from farm activities.

**Figure 14:**
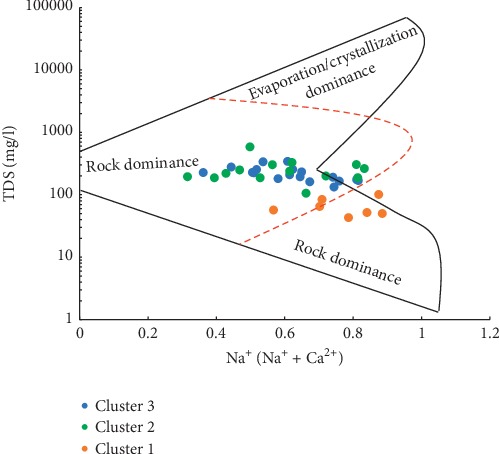
Gibbs diagram showing the main sources of variation in groundwater chemistry in the district.

Gibbs [68] diagram, for the three main clusters from the HCA, corroborates the main ions in the groundwater in the district, resulting mainly from the interaction of groundwater and rock/soil material as compared to other sources such as precipitation and evaporation (Figure 14). However, water samples in Cluster 1 from the HCA in the Gibbs diagram show the predominance of precipitation influencing the hydrochemistry in these portions of the district. This reinforces the assertion that these areas are mainly recharge zones dominated by fresh water types of meteoric origin. Figure 14 does not necessarily imply the absence of the impacts of evaporation on groundwater chemistry; it, however, suggests that evaporation does not significantly influence most of the major ions in groundwater across the district as compared to the other two factors based on the Gibbs [68] diagram.

To gain further understanding of the origin of the groundwater, biplots of the major ions which readily dissolve or react with other ions in groundwater have been plotted. The major cations of any groundwater type are usually dominated by Mg^2+^, Ca^2+^, and Na^+^, which are thought to be associated with the weathering and dissolution of minerals such as silicate, carbonate, and sulphate minerals and many more (equations (9)–(13)) [69, 70].

(9) $$\begin{array}{c} {\text{CO}}_{2}+{\text{H}}_{2}\text{O}⇌{\text{ H}}_{2}{\text{CO}}_{3}\left(\text{carbonic acid}\right) \end{array}$$

(10) $$\begin{array}{c} {\text{CaCO}}_{3}{\;+\text{ H}}_{2}{\text{CO}}_{3}⇌{\text{ Ca}}^{2+}{\;+2\text{HCO}}_{3-}\left(\text{calcite dissolution}\right) \end{array}$$

(11) $$\begin{array}{c} \text{CaMg}{\left({\text{CO}}_{3}\right)}_{2}\left(\text{dolomite}\right)+2\text{H}2\text{CO}3\;⇌{\text{ Ca}}^{2+}+{\text{Mg}}^{2+}+{4\text{HCO}}_{3-}\left(\text{dolomite dissolution}\right) \end{array}$$

(12) $$\begin{array}{c} {\text{H}}_{2}\text{O}+{\text{CaSO}}_{4}\cdot {\;2\text{H}}_{2}\text{O}⇌{\text{Ca}}^{2+}+{\text{SO}}_{4}^{2^{-}}+{3\text{H}}_{2}\text{O gypsum dissolution} \end{array}$$

(13) $$\begin{array}{c} 2{\text{NaAl}}_{2}{\text{Si}}_{3}{\text{O}}_{8}\left(\text{albite}\right)+2{\text{H}}_{2}{\text{CO}}_{3}+9{\text{H}}_{2}\text{O }⇌{\text{Al}}_{2}{\text{Si}}_{2}{\text{O}}_{5}{\left(\text{OH}\right)}_{4}\left(\text{Kaolinite}\right) \end{array}$$

**Figure 15:**
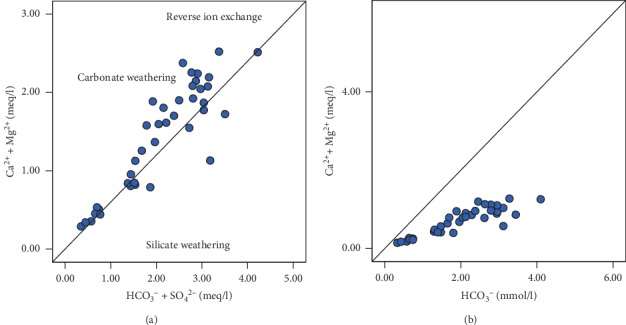
Biplots of (a) Ca^2+^+Mg^2+^ versus SO_4_^2−^ + HCO_3_^−^ and (b) Ca^2+^+Mg^2+^ versus HCO_3_^−^ showing the main sources of ions in groundwater chemistry.

A plot of Ca^2+^+Mg^2+^ versus SO_4_^2−^ + HCO_3_^−^ gives more insight into the weathering processes that led to the release of these ions in solution (Figure 15(a)). Samples below the equiline might have resulted from the weathering of silicate minerals, whereas samples above the equiline could be from carbonate mineral weathering of gypsum, calcite, and/or dolomite (Figure 15(a)). In such cases, carbonic acid from atmospheric reactions with water dissolves carbonate minerals which release Ca^2+^ and Mg^2+^ in solution (equations 9 to 11). The high concentration of Ca^2+^+Mg^2+^ relative to SO_4_^2−^ + HCO_3_^−^ is also attributable to reverse ion exchange, since the ratio is not exactly a 1 : 2.5 [71].

The origin of calcium and magnesium could also be understood by the plot of Ca^2+^+Mg^2+^ versus HCO_3_^−^ (Figure 15(b)). A molar ratio value of (Ca^2+^+Mg^2+^)/HCO_3_^−^ close to 0.5 suggests carbonate/silicate mineral weathering, as the main source of Mg^2+^ and Ca^2+^ in groundwater, influenced mainly by carbonic acid [72]. Some samples, however, fall above this 0.5 ratio which cannot be attributed to the depletion of HCO_3_^−^, since HCO_3_^−^ increases in the district from recharge areas to discharge zones, with a corresponding rise in pH, therefore suggesting ion exchange as the controlling factor in these samples' hydrochemistry [73].

**Figure 16:**
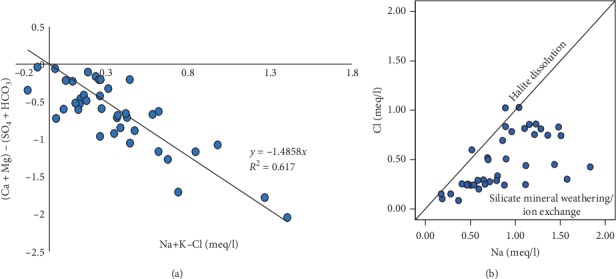
Biplot of (a) (Ca^2+^+Mg^2+^)–(SO_4_^2−^ + HCO_3_^−^) versus Na^+^+K^+^–Cl^−^ and (b) Na^+^ versus Cl^−^ suggesting ion exchange and silicate weathering in the hydrochemistry of the study area.

**Figure 17:**
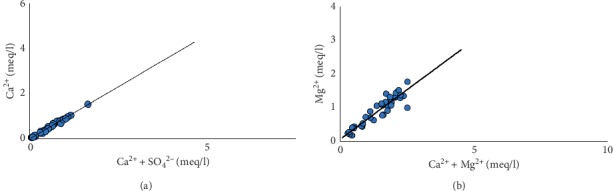
Biplot suggesting (a) gypsum weathering and (b) dolomite weathering.

Figure 16(a) confirms that some levels of ion exchange between Ca^2+^, Na^+^, and Mg^2+^ as a contributory factor in the hydrochemistry as a plot of (Ca^2+^+Mg^2+^)–(SO_4_^2−^ + HCO_3_^−^) versus Na^+^+K^+^–Cl^−^ have been used to assess this phenomenon [7]. A plot of these two indices which yields a slope of −1, in which the samples were plotted away from the origin, suggests the likelihood of significant impacts of ion exchange in the groundwater system (similar observations have been made in northern Ghana and within the Voltaian [7, 74]), whereas a slope which deviates significantly from −1 and clusters in the origin suggests otherwise. The nature of the ion exchange is suggested to be dominated by reverse cation exchange and probably the weathering of Na^+^-rich mineral, from the plot of Na^+^ versus Cl^−^ (Figure 16(b)). This assertion corroborates that of the Durov plot (Figure 13) and is partly based on the fact that halite dissolution does not account for the Na^+^ ions in the groundwater and the dominance of Na^+^ ions over Cl^−^ ions in the 1 : 1 plot. The chemical processes are further understood by the possible weathering of gypsum (Figure 17(a)) and dolomite (Figure 17(b)). The dissolution of dolomite which releases Mg^2+^ and Ca^2+^ in solution (equation (11)) appears to be a significant carbonate weathering process in the groundwater system, whereas gypsum dissociation results in Ca^2+^ and SO_4_^2−^ especially in recharge zones, where the bedrock is exposed [62].

### 4.3. Chemical Groundwater Quality Assessment for Domestic Purposes

Access to the quality water supply is a basic human right and an important tool for socioeconomic development for many countries since it goes a long way to reduce adverse health impacts and health costs [59]. Since groundwater in the district is mainly used for drinking and other domestic purposes, some chemical parameters with critical health implications from the analysis are being examined further to ascertain the groundwater suitability for such domestic purposes. pH distribution in the district is characteristically low and ranges from 5.20 to 7.60. The lowest values occur in and around Tula, Ningo, and Bingu in the southeastern portions of the district, underlain mainly by the Voltaian, whereas the highest values occur in Tongo and Datuku areas in the central and northeastern parts of the district.

Low pH values in groundwater arise mainly from carbonic acid from precipitation; however, the oxidation of sulphur and nitrogen compounds, dissociation of humus acids, the hydrolysis and oxidation of ferrous iron, and cation exchange are all factors which control pH variation in groundwater [75]. Acidic conditions in groundwater could also result from agrochemicals, especially the use of ammonium sulphate as fertilizer. Although pH has no direct health impacts on consumers, extreme values can affect the palatability of water, also cause corrosion of distribution systems, and enhance the solubility of most minerals and heavy metals in groundwater which might have adverse health impacts. Similarly, total hardness (TH) (as CaCO_3_) showed high levels above recommended WHO [20] values for portable and domestic water usage. Generally, groundwater in the district is a hard water type with only about 26% being soft water. Although WHO [59] reports an inverse relationship between TH and cardiovascular diseases in areas with hard water, TH above 200 mg/l is likely to cause scales deposition in water treatment systems, storage systems and pipes, and excessive soap consumption, since it does not lather easily, and subsequent scum formation [59].

**Figure 18:**
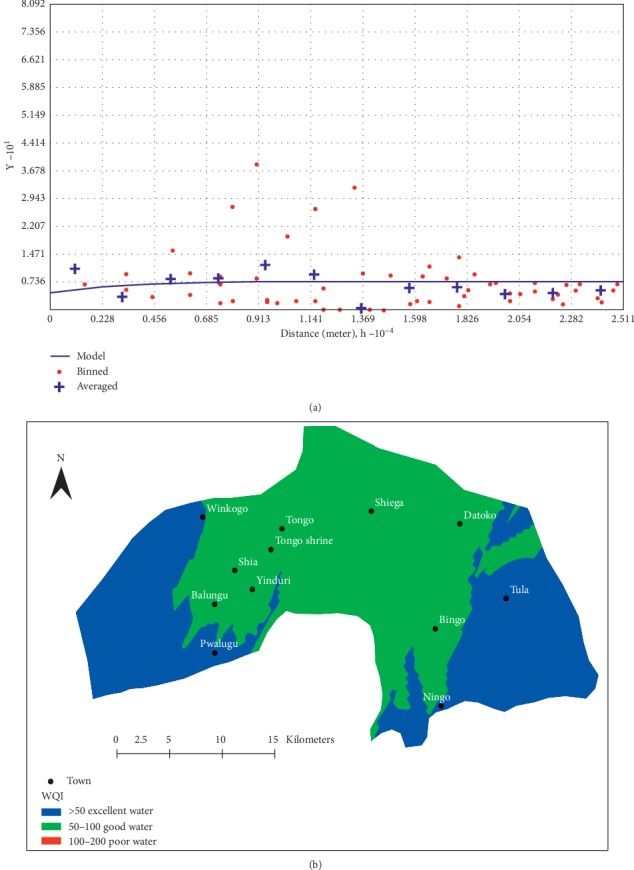
(a) Semivariogram model for WQIs and (b) spatial distribution of water quality indices.

The water quality index approach [54] adopted in this study suggests that groundwater in the district is of acceptable quality as this classification method places all samples within good to excellent water category, except for one sample. About 48% of the water samples each fell within excellent and good categories whereas the remaining 2%, which is just one sample, fell within the poor category. The poor water is a sample from Sheagar, with high levels of Pb and NO_3_^−^ which has resulted in its unwholesomeness. This is most likely a localised problem which might have resulted from the leaching of dissolved metals and/or agrochemicals into this well, since this particular well is a hand-dug well and shallow in depth. Furthermore, the WQIs were log-transformed and used in a GIS environment to generate a spatial prediction map for the water quality variations across the district. Prior to that, a variogram based on ordinary kriging was performed to visualise the spatial autocorrelation of the dataset. Various theoretical semivariogram models were tried on the dataset to attain the best fit with the aim of achieving the least root mean square residual error for the chosen model. An exponential model variogram with a range of 25106 m, a search direction of 12^o^, a sill of 0.033, and a nugget of 0.03 (Figure 18(a)) was used to generate the spatial prediction map (Figure 18(b)) to show the WQIs across the district for domestic purposes. The nugget suggests variations in water quality at distances shorter than the lag distance of 2092 m, which could be as a result of local anthropogenic influences and/or instrument and measurement errors.

From the spatial prediction map (Figure 18(b)), it is clear that groundwater resources are generally of great chemical quality for domestic purposes. Groundwater around the southeastern and southwestern portions of the district proves to be of the best quality for domestic purposes. Communities around such areas include Ningo, Tula, Bingu, Pwalugu, Balungu, and Shia. Although the Tula, Ningo, and Bingo areas are characterised by low pH values, that seems not to affect the overall water quality for domestic uses. Hence, the illegal small-scale gold mining activities around Bingu and Tula areas seem also not to affect the quality of groundwater in the area. TDSs seem to positively affect the quality of the water in the district since the best WQIs occur around the discharge zones with high TDS values.

Generally, the quality of the groundwater for domestic usage deteriorates as one moves towards the extreme north of the district. About 71% of the samples had Pb concentrations between 0.02 mg/l and 0.03 mg/l which exceed the WHO [20] recommended standards of 0.01 mg/l for drinking water. These samples are mainly from the central and northern portions of the district and, therefore, contribute to the worsening quality of groundwater in such areas (Figure 18(b)). A well from Sheagar recorded elevated levels of nitrate and phosphate which suggest the leaching of agrochemicals or organic manure from farms or domestic wastewater from homes that drain into the catchment of the well, which happens to be a shallow hand-dug well as well, thereby affecting its quality.

### 4.4. Assessment of Groundwater Quality for Irrigation

The groundwater is also being assessed to examine its suitability for irrigated agriculture which could augment the predominantly rain-fed agriculture; this would go a long way to improve the livelihood of indigents as well as provide all-year-round employment for the youth, which would also reduce rural-urban migration that characterise the district. Various crops have different tolerance levels for the different chemical parameters in water. Similarly, the various chemical parameters affect various crops differently at different concentrations and conditions. Some of the water quality parameters are also known to affect soil structure and permeability which goes a long way to affect its productivity and yield and, by extension, the quality and yield of crops. As such, an evaluation of groundwater quality for irrigation purposes which estimates chemical parameters and indices of chemicals which are likely to have detrimental impacts on the soil and crops when found in water used for crop irrigation is vital for healthy and productive irrigated agriculture.

**Figure 19:**
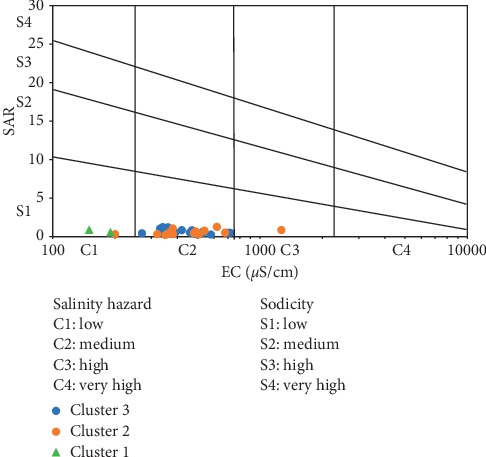
Groundwater quality classification in the district for irrigation [47].

The United States Salinity Laboratory [47] diagram which plots SAR versus EC on a semilogarithmic scale has been used in this study to assess irrigation water quality. The diagram categorises irrigation water in the ranges of low to very high sodicity on the SAR axis and low to very high salinity hazard on the EC axis. Based on this categorisation, all the groundwater samples in the district fell in the low sodicity category (S1), whereas 8% and 89% fell in low and medium salinity (C1-C2) category, respectively, with a lone sample falling in the S1-C3 category (Figure 19). Therefore, about 97% of the water samples present excellent quality for irrigation purposes and may be used for such purposes without posing any hazard to the soil or crops. This assertion is, however, dependent on the initial soil conditions, such that soils with excess sodium and/or salinity must be treated prior to irrigation with any water type. The lone sample which is plotted in the S1-C3 category may also be used for irrigation, but in a well-drained soil as a result of the high salinity hazard associated with this water type, to prevent restricted flow and subsequent accumulation of salts in the root zone of crops which leads to salinity and permeability problems [55, 74].

**Figure 20:**
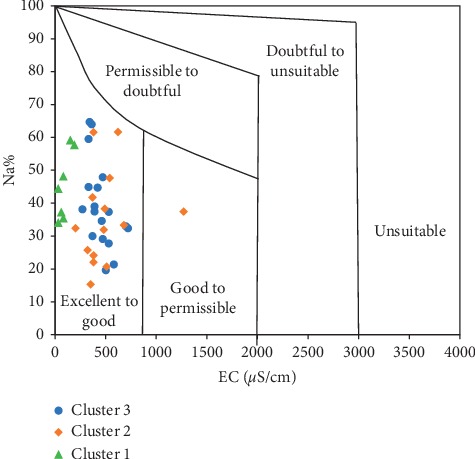
Groundwater quality assessment using Wilcox [46] diagram.

The Wilcox [46] diagram (Figure 20), on the other hand, presents the categories of water types in the district for irrigation purposes, depending on the corresponding combination of sodium percent and salinity hazard. All groundwater samples in the district were plotted in the excellent to good category, except for one sample which fell within good to permissible range. Although the water exhibits relatively high sodium percent, Cluster 1 samples plot the lowest salinity values, confirming their freshness in terms of flow regime. Generally, the plots (Figure 20) imply that groundwater across the district is of excellent quality and may be used for irrigation without posing any threat to the soil or crops, in which assertions corroborate those made by the USSL diagram.

Irrigation has a long-term effect of affecting the permeability of the soil. Doneen [76] diagram has been used to further classify the water into three classes based on sodium and bicarbonate hazard on irrigated soil. Bicarbonate hazard arises from soils with deficient iron content and certain micronutrients relevant for healthy plant growth and development. As such the permeability index (PI) measures the relative concentrations of sodium and bicarbonate to calcium, magnesium, and sodium based on the following formulation:

(14) $$\begin{array}{c} \text{PI}=\frac{\text{Na}+\sqrt{\text{HCO}3}}{\text{Na}+\text{Ca}+\text{Mg}}\times 100\text{,} \end{array}$$

where ion concentrations are all in meq/l.

**Figure 21:**
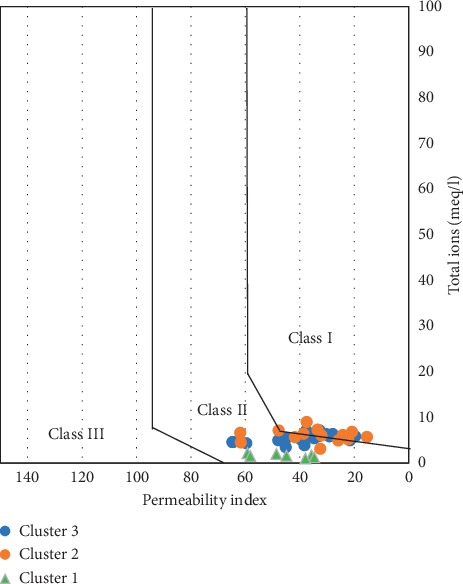
Doneen diagram for Talensi District.

The Doneen diagram categorised groundwater in the district as mainly Class II and Class I types (Figure 21) with none falling within Class III, where Class I and Class II are, respectively, excellent and good permeability for irrigation purposes. The Class II water type comprises about 50% of the water samples and is mainly samples from Cluster 1, which contains relatively high levels of sodium as compared to the other major cations and, therefore, suggests a comparatively higher sodium hazard.

## 5. Conclusion

The results of this study provide insight into the main controls of groundwater chemistry in Talensi District and its suitability for various uses. Water quality indices (WQIs) calculated in this study to assess the suitability of groundwater for domestic purposes suggest that groundwater in the district is of acceptable quality for such purposes, as this classification method placed 96% of the samples within good to excellent water category. Generally, the quality of the groundwater for domestic usage deteriorates as one moves towards the extreme north of the district, whereas waters in the extreme east and west present the best quality.

Multivariate statistical analysis and conventional graphical methods applied to groundwater samples suggest silicate and carbonate mineral weathering as the main control of groundwater chemistry in the district, with reverse ion exchange also playing a role. High nitrate and lead levels have been associated with agrochemicals and wastewater from farms and homes. Furthermore, Q-mode cluster analysis identifies three zones of groundwater flow regimes, in which the water evolves from a Na + K–Mg–HCO_3_ dominant fresh water type in Cluster 3, identified as intermediary flow zones, to Mg–Na + K–HCO_3_ fresh water type in Cluster 2, designated as discharge zones with corresponding increasing mineralisation of the groundwater. All three clusters present excellent quality for irrigation purposes, with Cluster 1 samples being even more so, based on the sodium-based/related assessments adopted in this study.

## Figures and Tables

**Table 1:** Standards, weights, and relative weights used for WQI computation.

Chemical parameter	Objective to be met (*Si*) (mg/l)	Weight (*wi*)	Relative weight (Wi)
pH	7.5	5	0.102
TDS	500	4	0.0816
TH	200	4	0.0816
Ca^2+^	200	2	0.0408
Mg^2+^	150	2	0.0408
Na^+^	200	2	0.0408
K^+^	30	2	0.0408
CI^−^	250	3	0.0612
SO_4_^2−^	250	3	0.0612
NO_3_^−^	50	5	0.102
PO_4_^3−^	0.7	4	0.0816
F^−^	1.5	5	0.102
Pb	0.01	5	0.102
Fe	0.3	3	0.0612

**Table 2:** Water quality index (WQI) categorisation [54].

WQI	Category
<50	Excellent water
50–100	Good water
100–200	Poor water
200–300	Very poor water
>300	Water unsuitable for drinking

**Table 3:** Summary statistics of major physicochemical parameters used for the study.

Major parameter	Minimum	Maximum	Mean	Std. deviation
Temp (°C)	28.7	34.4	31.6	1.6
EC (uS/cm)	30	1270	403.85	229.22
pH	5.20	7.60	6.80	0.60
TDS	43	584	204	102.20
Ca^2+^	0.96	30.36	10.68	6.45
Mg^2+^	2.14	21.23	10.89	5.19
Na^+^	4.06	42.15	20	9.34
K^+^	0.04	1.10	0.41	0.27
TH	72	412	204.95	81.99
HCO_3_^−^	20	250	125.50	58.24
CI^−^	3.02	36.50	17	9.91
SO_4_^2−^	<0.01	9.76	3	2.34
NO_3_^−^	<0.01	2.98	0.46	0.70
PO_4_^3−^	<0.01	8.59	0.37	1.36
Pb	<0.01	0.04	0.02	0.006
F^−^	<0.01	1.47	0.37	0.40
SAL	<0.01	0.50	0.10	0.09

**Table 4:** Pearson's correlation matrix between water quality parameters.

	pH	EC	TDS	SAL	TH	Ca^2+^	Mg^2+^	Na^+^	K^+^	HCO_3_^−^	Cl^−^	SO_4_^2−^	NO_3_^−^	PO_4_^3−^	F^−^
pH	1.00														
EC	**0.79**	1.00													
TDS	**0.82**	**0.96**	1.00												
SAL	**0.57**	**0.76**	**0.83**	1.00											
TH	**0.61**	**0.68**	**0.74**	**0.66**	1.00										
Ca^2+^	**0.84**	**0.84**	**0.88**	**0.67**	**0.70**	1.00									
Mg^2+^	**0.80**	**0.73**	**0.76**	**0.51**	**0.57**	**0.76**	1.00								
Na^+^	**0.67**	**0.77**	**0.75**	**0.62**	**0.52**	**0.54**	0.41	1.00							
K^+^	0.09	0.09	0.16	0.08	0.25	0.12	0.33	0.00	1.00						
HCO_3_^−^	**0.94**	**0.86**	**0.90**	**0.69**	**0.66**	**0.88**	**0.89**	**0.67**	0.24	1.00					
Cl^−^	0.46	**0.72**	**0.67**	**0.56**	**0.59**	**0.52**	0.33	**0.73**	0.03	0.45	1.00				
SO_4_^2−^	0.36	0.40	0.36	0.26	−0.03	0.41	0.29	0.29	−0.26	0.37	0.07	1.00			
NO_3_^−^	−0.15	−0.04	−0.12	−0.04	0.03	0.05	−0.20	0.07	−0.09	−0.16	0.12	−0.10	1.00		
PO_4_^3−^	−0.05	−0.12	−0.07	−0.07	0.08	−0.01	0.02	−0.07	0.05	−0.05	0.06	−0.34	0.21	1.00	
F^−^	0.38	0.33	0.32	0.24	0.22	0.37	0.29	0.21	−0.11	0.38	0.03	**0.59**	0.15	−0.13	1.00

**Table 5:** Final factor loadings for the water quality parameters.

	Component		
	1	2	3
EC	0.940	0.163	
HCO_3_^−^	0.921	0.165	0.277
pH	0.886	0.205	0.177
Ca^2+^	0.879	0.179	0.12
Na+	0.804		−0.32
Mg^2+^	0.796		0.453
Cl^−^	0.737	−0.19	−0.41
SO_4_^2−^	0.296	0.821	
F^−^	0.277	0.698	
PO_4_^3−^		−0.571	
K^+^	0.196	−0.456	0.643
NO_3_^−^		−0.315	−0.64

**Table 6:** Total variance explained.

Component	Initial eigenvalues			Extraction sums of squared loadings			Rotation sums of squared loadings		
	Total	% of variance	Cumulative %	Total	% of variance	Cumulative %	Total	% of variance	Cumulative %
1	5.596	46.634	46.634	5.596	46.634	46.634	5.319	44.324	44.324
2	1.736	14.464	61.098	1.736	14.464	61.098	1.967	16.394	60.718
3	1.393	11.611	72.709	1.393	11.611	72.709	1.439	11.991	72.709
4	0.967	8.061	80.769						
5	0.744	6.204	86.973						
6	0.557	4.642	91.615						
7	0.347	2.891	94.507						
8	0.318	2.646	97.152						
9	0.179	1.496	98.648						
10	0.105	0.876	99.524						
11	0.044	0.367	99.891						
12	0.013	0.109	100.000						

## Data Availability

The hydrochemical data used to support the findings of this study have been deposited in the Mendeley Data Repository by Chegbeleh et al. [77].
